# Sending-or-not-sending twin-field quantum key distribution in practice

**DOI:** 10.1038/s41598-019-39225-y

**Published:** 2019-02-28

**Authors:** Zong-Wen Yu, Xiao-Long Hu, Cong Jiang, Hai Xu, Xiang-Bin Wang

**Affiliations:** 1 State Key Laboratory of Low Dimensional Quantum Physics, Tsinghua University, Beijing, 100084 People’s Republic of China; 2 Data Communication Science and Technology Research Institute, Beijing, 100191 People’s Republic of China; 3 Synergetic Innovation Center of Quantum Information and Quantum Physics, University of Science and Technology of China, Hefei, Anhui 230026 People’s Republic of China; 4 Shandong Academy of Information and Communication Technology, Jinan, 250101 People’s Republic of China; 5 Department of Physics, Southern University of Science and Technology, Shenzhen, 518055 People’s Republic of China

## Abstract

Recently, the twin field quantum key distribution (TF-QKD) protocols have been investigated extensively. In particular, an efficient protocol for TF-QKD with sending or not sending the coherent state has been given in. Here in this paper, we present results of practical sending-or-not-sending (SNS) twin field quantum key distribution. In real-life implementations, we need consider the following three requirements, a few different intensities rather than infinite number of different intensities, a phase slice of appropriate size rather than infinitely small size and the statistical fluctuations. We first show the decoy-state method with only a few different intensities and a phase slice of appropriate size. We then give a statistical fluctuation analysis for the decoy-state method. Numerical simulation shows that, the performance of our method is comparable to the asymptotic case for which the key size is large enough. Our method can beat the PLOB bound on secret key capacity. Our results show that practical implementations of the SNS quantum key distribution can be both secure and efficient.

## Introduction

Quantum key distribution (QKD) allows two parties, Alice and Bob, to share unconditional secret keys based on the laws of quantum physics^1–6^, even in the presence of an eavesdropper, Eve. However, in real-life implementations of QKD, its practical security is still questionable due to the device imperfections, such as the imperfect source^7–9^ and detectors. Fortunately, by using the decoy-state method^10–25^, it has been shown that the unconditional security of QKD can still be assured with an imperfect single-photon source. To avoid the detector side channel attacks, the measurement-device-independent QKD (MDI-QKD) was proposed^26,27^. The decoy-state MDI-QKD can remove all detector side-channel attacks with imperfect single-photon sources^28–33^.

With the developments^10–44^ in both theory and experiment, QKD is more and more hoped to be extensively applied in practice, though there are barriers for doing so. Among them, the transmission loss of photons for long distance QKD has become the major obstacle in practical implementations. Very recently, a milestone breakthrough was made under the name of twin-field quantum key distribution (TF-QKD)^45^ for long distance QKD with a key rate scales in square root of channel transmittance. To offer the information-theoretic-security, a number of upgraded variants were then proposed^1,46–48^. In particular, an efficient protocol for TF-QKD with sending or not sending the coherent state has been given in ref.^1^. In the sending-or-not-sending (SNS) protocol^1^, Alice and Bob do not take post selection for the bits in *Z* basis (signal pulses) and hence the traditional calculation formulas directly apply. Also, it is fault tolerant to misalignment errors in the long distance single-photon interference.

In practice, we need consider the situations with a few different intensities rather than infinite number of different intensities, a phase slice of appropriate size and the statistical fluctuations. It should be interesting to see whether the advantage in the twin-field QKD still holds with these conditions in practice. In this paper, we proceed further and analyse the performance of the SNS TF-QKD under the above real-life assumptions and we show that the advantage in distance and key rate still holds..

First, we reveal the decoy-state method with only a few different intensities and a phase slice of appropriate size to estimate the lower bound of the yield and the upper bound of the phase-flip error rate for the single-photon state. Furthermore, we also need to consider the statistical fluctuations. In order to improve the results, the instances for basis unmatched are also used to estimate the lower bound of the yield for the single-photon state, such as in Eq. (1).

## Results

### The decoy-state method with a few different intensities and a phase slice of appropriate size

In the four-intensity decoy-state SNS protocol, Alice and Bob randomly choose the *X*-window (decoy pulses) and *Z*-window (signal pulses) to send or not to send a phase-randomized coherent pulse to an untrusted party, Charlie, who is expected to perform interference measurement. The protocol is detailed below.

1. Alice and Bob repeat Steps 2–3, *N* times. All the public announcements by the legitimate users Alice and Bob are done over an authenticated channel.
2. Alice and Bob randomly choose *X*-window and *Z*-window with probabilities *p*_*X*_ and 1−*p*_*X*_ respectively. Alice (Bob) prepares and sends the decoy pulses in her (his) *X*-window. Explicitly she (he) randomly choose one of three sources ${\rho }_{{\alpha }_{i}}$ with probability *p*_*i*_ for *i* = 0, 1, 2, where ${\rho }_{{\alpha }_{0}}=\mathrm{|0}\rangle \langle \mathrm{0|}$ is the vacuum source, ${\rho }_{{\alpha }_{1}}$ and ${\rho }_{{\alpha }_{2}}$ are two phase-randomized coherent sources with intensity *μ*_1_ and *μ*_2_ (*μ*_1_ < *μ*_2_) respectively. In *Z*-window, Alice (Bob) puts down a bit value 1 and prepares and sends the phase-randomized coherent state ${\rho }_{{\alpha }_{z}}$ with probability *p*_*z*_, or puts down a bit value 0 and sends nothing else, i.e., sends the vacuum pulse with probability 1−*p*_*z*_.
3. Charlie measures the incoming signals and records which detector clicks. When the quantum communication is over, he publicly announces all the information about the detection event. The situation when one and only one detector (detector 0 or detector 1) makes a count is denoted as an effective event. Alice and Bob collect all the data with effective events and discard all the others.
4. Alice and Bob announce the basis information (*X*-window or *Z*-window) firstly. Then they announce the bit values and phase information corresponding to the effective events when Alice or Bob choose *X*-window. With these information, Alice and Bob obtain the observable *N*_*jk*_(*j*, *k* = 0, 1, 2, *z*) being the number of instances when Alice and Bob send state ${\rho }_{{\alpha }_{j}}$ and ${\rho }_{{\alpha }_{k}}$ respectively. Correspondingly, the lowercases *n*_*jk*_ are used to denote the number of effective events. The yields can be defined as *S*_*jk*_ = *n*_*jk*_/*N*_*jk*_. Explicitly, we have *N*_11_, *N*_22_ and *N*_*zz*_ are the number of instances when Alice and Bob send state ${\rho }_{{\alpha }_{1}}$, ${\rho }_{{\alpha }_{2}}$ and ${\rho }_{{\alpha }_{z}}$ respectively. Furthermore, In order to improve the results, the instances for basis unmatched are also considered and
  1 $$\begin{array}{rcl}{N}_{00} & = & {p}_{0}^{2}{N}_{X}+2{p}_{0}\mathrm{(1}-{p}_{z}){N}_{XZ},\\ {N}_{01} & = & {N}_{10}={p}_{0}{p}_{1}{N}_{X}+\mathrm{(1}-{p}_{z}){p}_{1}{N}_{XZ},\\ {N}_{02} & = & {N}_{20}={p}_{0}{p}_{2}{N}_{X}+\mathrm{(1}-{p}_{z}){p}_{2}{N}_{XZ},\end{array}$$
  where *p*_0_ = 1−*p*_1_−*p*_2_ is the probability to send a vacuum pulse in *X*-window, ${N}_{X}={p}_{X}^{2}N$ is the number of instances when both Alice and Bob choose *X*-window and *N*_*XZ*_ = *p*_*X*_(1−*p*_*X*_)*N* is the number of instances when Alice chooses *X*-window and Bob chooses *Z*-window.
5. Define two sets ${C}_{{{\rm{\Delta }}}^{+}}$ and ${C}_{{{\rm{\Delta }}}^{-}}$ that contain the instances when both Alice and Bob send ${\rho }_{{\alpha }_{1}}$ in *X*-window with the phase information *θ*_*A*_ and *θ*_*B*_ falling into the slice |*θ*_*A*_−*θ*_*B*_| ≤ Δ/2 and |*θ*_*A*_−*θ*_*B*_−*π*| ≤ Δ/2 respectively. The number of instances in ${C}_{{{\rm{\Delta }}}^{\pm }}$ are ${N}_{11}^{{{\rm{\Delta }}}^{\pm }}=\frac{{\rm{\Delta }}}{2\pi }{N}_{11}$. The number of effective events corresponding to ${C}_{{{\rm{\Delta }}}^{\pm }}$ are denoted by ${n}_{11}^{{{\rm{\Delta }}}_{0}^{\pm }}$ and ${n}_{11}^{{{\rm{\Delta }}}_{1}^{\pm }}$ for detector 0 and detector 1 respectively.
6. With these observables, Alice and Bob can estimate the lower bound of *n*_1_ and the upper bound of ${e}_{1}^{ph}$ by using the decoy-state methods shown below. Then the post-processing can be performed and the final key length is

2 $${N}_{f}={n}_{1}\mathrm{[1}-H({e}_{1}^{ph})]-f{n}_{t}H({E}_{Z}),$$

where *N*_*f*_ is the number of final bits, *n*_1_ is the number of effective events caused by single-photon states in *Z*-basis when Alice decides sending while Bob decides not sending or Alice decides not sending while Bob decides sending, ${e}_{1}^{ph}$ is the phase-flip error rate for instances of *n*_1_, $H(p)=-\,p{\mathrm{log}}_{2}(p)-\mathrm{(1}-p){\mathrm{log}}_{2}\mathrm{(1}-p)$ is the binary entropy function, *f* is the correction efficiency, *n*_*t*_ is the number of effective events when both Alice and Bob choose *Z*-window and *E*_*Z*_ is the corresponding bit-flip error rate.

Alternatively, we also have the equivalent formula for key rate per time window as shown in the section Methods.

In the above, for conciseness, we have omitted those mismatching time windows in a real protocol. For example, when Alice commits to a decoy window and Bob commits to a signal window. Although the events of these windows cannot be used for the final key distillation, the data for heralded events from these time windows can be used in the decoy-state analysis. The bit value encoding is defined by Alice or Bob’s decision on sending or not-sending in a signal window. As shown in ref.^1^, we can relate the bit values with local ancillary states in the virtual protocol. Clearly, there isn’t any definition confusion^47^ in the SNS protocol^1^.

A tricky point in the SNS protocol is that the traditional decoy-state method can still work. In this protocol, the random phase information of *Z*-windows are never announced therefore we can regard pulses of *Z*-basis as classical mixture of different photon number states properly. Note that, very importantly, the random phase information in *Z* windows can never been announced because otherwise, the elementary concepts such as the number of single-photon counts are *illy* defined. But, as shown in details in ref.^1^, the random phase information in *X*-windows can be post announced. Because we only want to verify the phase-flip error rate of *Z* windows. The phase-flip rate of *Z* windows is an objective fact, once it is verified, it is there. The post announced phase information does not change this objective facts because no matter how Eve takes action with the post announced information, the action is just Eve’s local action which can not make a difference to anything detectable to Alice and Bob.

### Numerical simulation

In this section, we present some results of the numerical simulation. In order to show the efficiency of our method, without any loss of generality, we focus on the symmetric case where the two channel transmissions from Alice to Charlie and from Bob to Charlie are equal. We also assume that Charlie’s detectors are identical, i.e., they have the same dark count rates and detection efficiencies, and their detection efficiencies do not depend on the incoming signals. The results for the asymmetric case will be considered in the coming work. We shall estimate what values would be probably observed in the normal cases by the linear models as previously. The values of the experimental parameters used in the simulations are listed in Table 1.

**Table 1:** List of experimental parameters used in numerical simulations.

*p*_*d*_	*η*_*d*_	*f*	*ε*	*e*_*a*_
1.0 × 10^−10^	50%	1.1	1.0 × 10^−10^	15%

*p*_*d*_: the dark count rate, *η*_*d*_: the detection efficiency of all detectors, *f*: the error correction inefficiency, *ε*: the security bound considered in the statistical fluctuation analysis, *e*_*a*_: the misalignment error.

**Figure 1:**
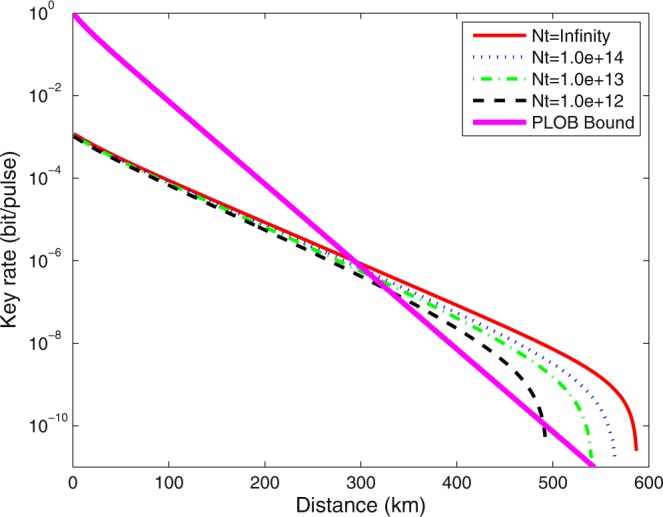
Optimal key rate (bits per pulse) as a function of the distance by 4-inensity decoy-state method. The asymptotic result is shown in the red solid line. The blue dotted line, the green dash-dot line and the black dashed line are the results with *N* = 10^14^, *N* = 10^13^ and *N* = 10^12^, respectively. The solid magenta thick line illustrates the PLOB bound.

**Figure 2:**
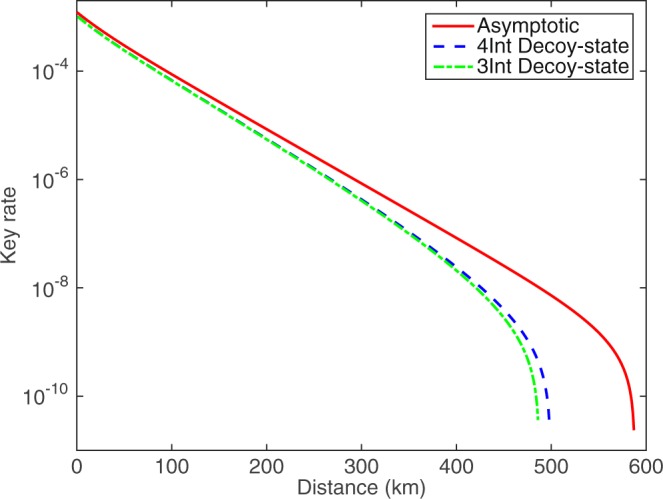
Optimal key rate (bits per pulse) as a function of the distance. The asymptotic result is shown in the red solid line. The blue dashed line and the green dash-dot line are the results for 4-intensity and 3-intensity decoy-state methods with *N* = 10^12^, respectively.

**Figure 3:**
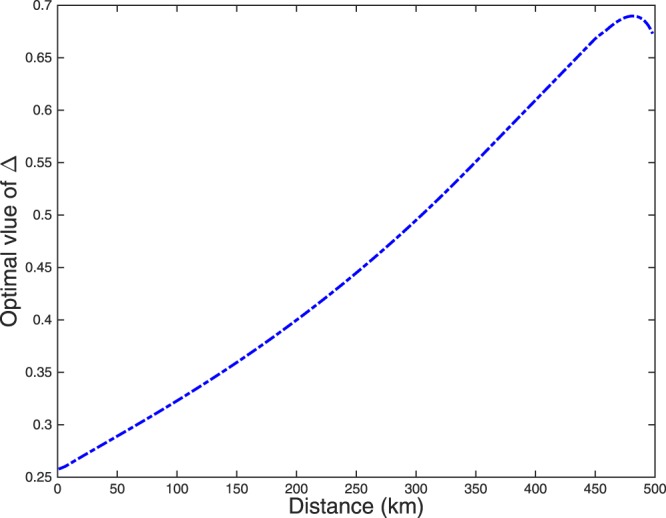
Optimal value of Δ (radians) corresponding to the optimal key rate by 4-intensity decoy-state method with *N* = 10^12^.

We optimize all parameters, *p*_*X*_, *p*_1_, *p*_2_ *p*_*z*_, *μ*_1_, *μ*_2_, *μ*_*z*_ and Δ by the method of full optimization. The results of optimized key rate with different *N* by four-inensity decoy-state method and the result with theoretical PLOB bound^49^ are shown in Fig. 1. In it, we use the red solid line to denote the asymptotic results with infinite number of pulses. The optimal key rate with *N* = 10^14^, *N* = 10^13^ and *N* = 10^12^ are shown by the blue dotted line, the green dash-dot line and the black dashed line respectively. The result with theoretical PLOB bound is plotted by the thick magenta solid line. The numerical simulations show that the finite-size SNS protocol can overcome the PLOB bound. In Fig. 2, we plot the final key rates by the four-intensity and the three-intensity decoy-state methods with *N* = 10^12^. We can see that the optimal key rates for the three-intensity decoy-state method is nearly equal to the results for the four-intensity decoy-state method when we are aim for practically useable key-rates (such as 10^−6^ per-pulse). In Fig. 3, we plot the optimal value of Δ for different distances with *N* = 10^12^ by four-inensity decoy-state method. With this, we know that the optimal value of Δ are changed with different communication distance between Alice and Bob. The optimal value of Δ monotonically increases, to reduce the impact of statistical fluctuations, until it reaches a peak where the optimal key rate becomes decreasing dramatically and the error rate has a greater impact on the key rate than the statistical fluctuation.

Also, according to the observed data there^36^, we use a linear loss model to estimate the actual loss in the experiment for 404 km of ultralow-loss optical fiber (0.16 dB/km). Assuming the same device parameters (*p*_*d*_ = 7.2 × 10^−8^, *η*_*d*_ = 0.5525, *f* = 1.16, *ε* = 10^−10^, *e*_*a*_ = 2% and *N* = 6.0 × 10^14^), we make the optimization by using our SNS protocol with the four-intensity decoy-state method shown above. We obtain a final key rate of 141 bit per second (bps), which is more than 4.4 × 10^5^ times higher than the reported experimental result, 3.2 × 10^−4^ bps. Similarly, assuming the same device parameters (*p*_*d*_ = 4.0 × 10^−11^, *η*_*d*_ = 0.5, *f* = 1.1, *ε* = 5.0 × 10^−11^, *e*_*a*_ = 2% and *N* = 2.178 × 10^14^) for 421 km of ultralow-loss optical fiber (0.17 dB/km) in ref.^50^, we obtain a final key rate of 2.62 × 10^3^ bit per second (bps), which is more than 1.05 × 10^4^ times higher than the reported experimental result, 0.25 bps.

## Discussion

In real setups of QKD, the practical situations with a few different intensities rather than infinite number of different intensities, a phase slice of appropriate size rather than infinitely small size and the statistical fluctuations must be considered. We first present the decoy-state method with a few different intensities and a phase slice of appropriate size. Then we show that the SNS protocol is a highly practical scheme even when the statistical fluctuations are considered. Numerical simulation shows that, the finite-size SNS protocol can exceed the PLOB bound. Our results show that practical implementations of the SNS TF-QKD can be both secure and efficient.

## Methods

### Decoy-state method analysis

In the protocol, Alice and Bob prepare and send the coherent pulses with randomized phase. The traditional formulas of decoy-state method can be applied directly. The coherent state whose phase is selected uniformly at random can be regard as a mixture of photon number states

3 $${\rho }_{{\alpha }_{j}}={e}^{-{\mu }_{j}}\sum _{n=0}^{\infty }\,\frac{{\mu }_{j}^{n}}{n!}|n\rangle \langle n|,\,\,(j=\mathrm{0,}\,\mathrm{1,}\,\mathrm{2,}\,z)$$

where *μ*_*j*_ = |*α*_*j*_|^2^ is the intensity of the coherent state |*α*_*j*_〉. Then the state when Alice decides not sending and Bob decides to send ${\rho }_{{\alpha }_{k}}$ is ${\rho }_{{\alpha }_{0}{\alpha }_{k}}={e}^{-{\mu }_{k}}{\sum }_{n=0}^{\infty }\,{\mu }_{k}^{n}/n\mathrm{!|0}n\rangle \langle 0n|$. With these convex forms, the lower bound of the yield of the state ${\rho }_{{z}_{01}}=\mathrm{|01}\rangle \langle \mathrm{01|}$ can be written into the following form^30^

4 $${s}_{{z}_{01}}\ge {s}_{{z}_{01}}^{L}=\frac{{\mu }_{2}^{2}{e}^{{\mu }_{1}}{S}_{01}-{\mu }_{1}^{2}{e}^{{\mu }_{2}}{S}_{02}-({\mu }_{2}^{2}-{\mu }_{1}^{2}){S}_{00}}{{\mu }_{1}{\mu }_{2}({\mu }_{2}-{\mu }_{1})},$$

where *S*_0*k*_ are the yield of the sources ${\rho }_{{\alpha }_{0}{\alpha }_{k}}$ for *k* = 1, 2, *S*_00_ is the yield when both Alice and Bob send the vacuum state. Similarly, the lower bound of the yield of the state ${\rho }_{{z}_{10}}=\mathrm{|10}\rangle \langle \mathrm{10|}$ can be written as

5 $${s}_{{z}_{10}}\ge {s}_{{z}_{10}}^{L}=\frac{{\mu }_{2}^{2}{e}^{{\mu }_{1}}{S}_{10}-{\mu }_{1}^{2}{e}^{{\mu }_{2}}{S}_{20}-({\mu }_{2}^{2}-{\mu }_{1}^{2}){S}_{00}}{{\mu }_{1}{\mu }_{2}({\mu }_{2}-{\mu }_{1})},$$

where *S*_*j*0_ are the yield of the sources ${\rho }_{{\alpha }_{j}{\alpha }_{0}}$ for *j* = 1, 2. With Eqs (4) and (5), the lower bound of the yield of single-photon state in *Z*-basis, i.e., the state ${\rho }_{1}^{Z}=\frac{1}{2}({\rho }_{{z}_{01}}+{\rho }_{{z}_{10}})$, has the following form

6 $${s}_{1}^{Z}\ge {s}_{1}^{Z}=\frac{1}{2}({s}_{{z}_{01}}^{L}+{s}_{{z}_{10}}^{L})\mathrm{.}$$

Note: Replacing the source *ρ*_2_ used in Eqs (4–6) with the source *ρ*_*z*_, we obtain the other lower bound of ${s}_{1}^{Z}$. With this replacement, source *ρ*_2_ is not used actually, then the four-intensity decoy-state method can be simplified to a three-intensity decoy-state method by taking *p*_2_ = 0. On the one hand, the three-intensity decoy-state method can be carried out easily in experiment. On the other hand, interested more in terms of practical key-rates instead of achieving the longest distance QKD possible (such as 10^−6^ per-pulse), the key rate of the three-intensity decoy-state method is only a little lower than (less than one percent for the cases discussed in the numerical simulation) the results for the four-intensity decoy-state method.

In the rest of this section, we show the formula to estimate the upper bound of ${e}_{1}^{ph}$ in Eq. (2) with the observable. The state of pulse pair when Alice sends the coherent state $|{\alpha }_{1}^{A}=\sqrt{{\mu }_{1}}{e}^{i{\theta }_{A}}\rangle$ and Bob sends the coherent state $|{\alpha }_{1}^{B}=\sqrt{{\mu }_{1}}{e}^{i{\theta }_{B}}\rangle$ is

7 $$\begin{array}{rcl}|{\alpha }_{1}^{A}\rangle |{\alpha }_{1}^{B}\rangle  & = & |\sqrt{{\mu }_{1}}{e}^{i{\theta }_{A}}\rangle |\sqrt{{\mu }_{1}}{e}^{i{\theta }_{B}}\rangle ={e}^{-{\mu }_{1}}\sum _{{k}_{1},{k}_{2}}\,\frac{{(\sqrt{{\mu }_{1}}{e}^{i{\theta }_{A}})}^{{k}_{1}}{(\sqrt{{\mu }_{1}}{e}^{i{\theta }_{B}})}^{{k}_{2}}}{\sqrt{{k}_{1}!}\sqrt{{k}_{2}!}}|{k}_{1}\rangle |{k}_{2}\rangle \\  & = & {e}^{-{\mu }_{1}}[\mathrm{|00}\rangle +\sqrt{{\mu }_{1}}({e}^{i{\theta }_{B}}\mathrm{|01}\rangle +{e}^{i{\theta }_{A}}\mathrm{|10}\rangle )+{\mu }_{1}(\frac{{e}^{2i{\theta }_{B}}}{\sqrt{2}}\mathrm{|02}\rangle \\  &  & +\,{e}^{i({\theta }_{A}+{\theta }_{B})}\mathrm{|11}\rangle +\frac{{e}^{2i{\theta }_{A}}}{\sqrt{2}}\mathrm{|20}\rangle )+\cdots ]\\  & = & {e}^{-{\mu }_{1}}[\mathrm{|00}\rangle +\sqrt{2{\mu }_{1}}{e}^{i{\theta }_{B}}|{\psi }_{1}^{{\delta }^{+}}\rangle +\frac{{(\sqrt{2{\mu }_{1}}{e}^{i{\theta }_{B}})}^{2}}{\sqrt{2}}|{\psi }_{2}^{{\delta }^{+}}\rangle +\cdots ]\\  & = & {e}^{-{\mu }_{1}}\sum _{n=0}^{\infty }\,\frac{{(\sqrt{2{\mu }_{1}}{e}^{i{\theta }_{B}})}^{n}}{\sqrt{n!}}|{\psi }_{n}^{{\delta }^{+}}\rangle .\end{array}$$

Similarly, we also have

8 $$|{\alpha }_{1}^{A}\rangle |-{\alpha }_{1}^{B}\rangle ={e}^{-{\mu }_{1}}\sum _{n\mathrm{=0}}^{\infty }\frac{{(-\sqrt{2{\mu }_{1}}{e}^{i{\theta }_{B}})}^{n}}{\sqrt{n!}}|{\psi }_{n}^{{\delta }^{-}}\rangle \mathrm{.}$$

In Eqs (7) and (8), the *n*-photon twin-field state $|{\psi }_{n}^{{\delta }^{\pm }}\rangle$ is defined as follows

9 $$|{\psi }_{n}^{{\delta }^{+}}\rangle =\frac{1}{\sqrt{{2}^{n}}}\sum _{m=0}^{n}\,\frac{\sqrt{n!}{e}^{im\delta }}{\sqrt{m!(n-m)!}}|m\rangle |n-m\rangle ,$$

10 $$|{\psi }_{n}^{{\delta }^{-}}\rangle =\frac{1}{\sqrt{{2}^{n}}}\sum _{m=0}^{n}\,\frac{{(-\mathrm{1)}}^{m}\sqrt{n!}{e}^{im\delta }}{\sqrt{m!(n-m)!}}|m\rangle |n-m\rangle ,$$

where *δ* = *θ*_*A*_−*θ*_*B*_. For the state in set ${C}_{{{\rm{\Delta }}}^{+}}$, the phase is selected uniformly at random in the slice with |*θ*_*A*_−*θ*_*B*_| ≤ Δ/2. Equivalently, in set ${C}_{{{\rm{\Delta }}}^{+}}$, the phase *θ*_*B*_ chosen by Bob in $|{\alpha }_{1}^{A}\rangle |{\alpha }_{1}^{B}\rangle$ can be regarded as uniformly distributed in [0, 2*π*) and the phase *θ*_*A*_ chosen by Alice satisfies the condition |*δ*| ≤ Δ/2. For any fixed value *δ*, we have

11 $$\begin{array}{rcl}{\rho }_{{\delta }^{+}} & = & \frac{1}{2\pi }{\int }_{0}^{2\pi }\,|{\alpha }_{1}^{A}\rangle |{\alpha }_{1}^{B}\rangle \langle {\alpha }_{1}^{A}|\langle {\alpha }_{1}^{B}|d{\theta }_{B}\\  & = & {e}^{-2{\mu }_{1}}\sum _{n\mathrm{=0}}^{\infty }\,\frac{{\mathrm{(2}{\mu }_{1})}^{n}}{n!}|{\psi }_{n}^{{\delta }^{+}}\rangle \langle {\psi }_{n}^{{\delta }^{+}}\mathrm{|.}\end{array}$$

Similarly, we also have

12 $$\begin{array}{rcl}{\rho }_{{\delta }^{-}} & = & \frac{1}{2\pi }{\int }_{0}^{2\pi }\,|{\alpha }_{1}^{A}\rangle |-{\alpha }_{1}^{B}\rangle \langle {\alpha }_{1}^{A}|\langle \,-\,{\alpha }_{1}^{B}|d{\theta }_{B}\\  & = & {e}^{-2{\mu }_{1}}\sum _{n=0}^{\infty }\,\frac{{\mathrm{(2}{\mu }_{1})}^{n}}{n!}|{\psi }_{n}^{{\delta }^{-}}\rangle \langle {\psi }_{n}^{{\delta }^{-}}|\mathrm{.}\end{array}$$

Considering the single-photon twin-field states in ${C}_{{\rm{\Delta }}}={C}_{{{\rm{\Delta }}}^{+}}\cup {C}_{{{\rm{\Delta }}}^{-}}$ for a fixed *δ*, we have

13 $${\rho }_{1}^{\delta }=\frac{1}{2}(|{\psi }_{1}^{{\delta }^{+}}\rangle \langle {\psi }_{1}^{{\delta }^{+}}|+|{\psi }_{1}^{{\delta }^{-}}\rangle \langle {\psi }_{1}^{{\delta }^{-}}|)={\rho }_{1}^{Z}.$$

So we know that the single-photon states in set *C*_Δ_ and in *Z*-basis have the same density matrices. The probability to emit a single-photon pulse from *C*_Δ_ is ${q}_{1}=2{\mu }_{1}{e}^{-2{\mu }_{1}}$. With this relations, we know that the bit-flip error rate of single-photon state in set *C*_Δ_ is equal to the phase-flip error rate ${e}_{1}^{ph}$ asymptotically. The bit-flip error yield for all instances in set *C*_Δ_ is

14 $${T}_{{\rm{\Delta }}}=\frac{1}{2}({T}_{{{\rm{\Delta }}}^{+}}+{T}_{{{\rm{\Delta }}}^{-}})=\frac{1}{2}({n}_{11}^{{{\rm{\Delta }}}_{1}^{+}}/{N}_{11}^{{{\rm{\Delta }}}^{+}}+{n}_{11}^{{{\rm{\Delta }}}_{0}^{-}}/{N}_{11}^{{{\rm{\Delta }}}^{-}})\mathrm{.}$$

where *T*_*k*_, *k* = Δ, Δ^+^, Δ^−^ is the proportion of wrong effective events in *C*_*k*_, e.g. in ${N}_{11}^{k}$. Attribute all the error to the single-photon state and the vacuum state, the upper bound of phase-flip error rate ${e}_{1}^{ph}$ can be estimated by

15 $${e}_{1}^{ph}\le {\bar{e}}_{1}^{ph}=\frac{{T}_{{\rm{\Delta }}}-\mathrm{1/2}{e}^{-2{\mu }_{1}}{S}_{00}}{2{\mu }_{1}{e}^{-2{\mu }_{1}}{\underline{s}}_{1}^{Z}},$$

where ${\underline{s}}_{1}^{Z}$ is the lower bound of ${s}_{1}^{Z}$ given in Eq. (6). Then the final key rate of per pulse can be calculated with

16 $$R={\mathrm{(1}-{p}_{X})}^{2}\mathrm{\{2}{p}_{z}\mathrm{(1}-{p}_{z}){a}_{1}{s}_{1}\mathrm{[1}-H({e}_{1}^{ph})]-f{S}_{Z}H({E}_{Z})\},$$

where *R* is the final key rate, ${a}_{1}={\mu }_{z}{e}^{-{\mu }_{z}}$ is the probability to emit a single-photon state from source *ρ*_*z*_, *s*_1_ is the yield of the single-photon state in *Z*-window when one party from Alice and Bob decides to send a signal states, ${e}_{1}^{ph}$ is the phase-flip error rate for those instance of *s*_1_, *S*_*Z*_ and *E*_*Z*_ are the yield and bit-flip error rate for instances when both Alice and Bob choose *Z*-window.

### Statistical fluctuation analysis

In the real protocol with finite data size, in order to extract the secure final key, we have to consider the effect of statistical fluctuations. To obtain the lower bound value for *s*_1_ and the upper bound value for ${e}_{1}^{ph}$ in the real protocol with finite *N*, one can implement the idea of ref.^25^, i.e., treating the averaged yield. Accordingly, define 〈*S*〉 as the mean value of yield *S*. Note that even though *S*_*jk*_(*j*, *k* = 0, 1, 2, *z*) are known values directly observed in the experiment, the mean values 〈*S*_*jk*_〉 are not. However, given the observed values *S*_*jk*_ and the corresponding number of pulse pairs, the confidence lower and upper limits of 〈*S*_*jk*_〉 can be calculated.

In order to obtain a tighter lower bound of $\langle {s}_{1}^{Z}\rangle$, we need introduce the following two yields

17 $${S}_{1}=\frac{1}{2}({S}_{01}+{S}_{10})=\frac{{n}_{01}}{2{N}_{01}}+\frac{{n}_{10}}{2{N}_{10}},$$

18 $${S}_{2}=\frac{1}{2}({S}_{02}+{S}_{20})=\frac{{n}_{02}}{2{N}_{02}}+\frac{{n}_{20}}{2{N}_{20}},$$

Replacing the observed yields with their mean values in Eqs (6) and (15), we can formulate the lower bound of $\langle {s}_{1}^{Z}\rangle$ and the upper bound of $\langle {e}_{1}^{ph}\rangle$ respectively. Explicitly, we have

19 $$\langle {s}_{1}^{Z}\rangle \ge \langle {\underline{s}}_{1}^{Z}\rangle =\frac{{\mu }_{2}^{2}{e}^{{\mu }_{1}}{\underline{S}}_{1}-{\mu }_{1}^{2}{e}^{{\mu }_{2}}{\bar{S}}_{2}-({\mu }_{2}^{2}-{\mu }_{1}^{2}){\bar{S}}_{00}}{{\mu }_{1}{\mu }_{2}({\mu }_{2}-{\mu }_{1})},$$

and

20 $$\langle {e}_{1}^{ph}\rangle \le \langle {\bar{e}}_{1}^{ph}\rangle =\frac{{\bar{T}}_{{\rm{\Delta }}}-\mathrm{1/2}{e}^{-2{\mu }_{1}}{\underline{S}}_{00}}{2{\mu }_{1}{e}^{-2{\mu }_{1}}\langle {\underline{s}}_{1}^{Z}\rangle }.$$

with

21 $${\underline{{\mathscr{U}}}}_{k}={{\mathscr{U}}}_{k}\mathrm{/(1}+{\delta }_{k}),\,{\bar{{\mathscr{U}}}}_{k}={{\mathscr{U}}}_{k}/\mathrm{(1}-{\delta ^{\prime} }_{k}),$$

for ${\mathscr{U}}=S,\,T$ and *k* = 00, 1, 2 and Δ. By using the multiplicative form of the Chernoff bound^29,33^, with a fixed failure probability *ε*, we can give an interval of 〈*S*_*k*_〉 with the observable *S*_*k*_, $[{\underline{S}}_{k},\,{\bar{S}}_{k}]$, which can bound the value of 〈*S*_*k*_〉 with a probability of at least 1−*ε*. Explicitly, with the function ${f}_{\delta }(x,\,y)=[-\,\mathrm{ln}(y\mathrm{/2)}+\sqrt{{(\mathrm{ln}(y\mathrm{/2))}}^{2}-8\,\mathrm{ln}(y\mathrm{/2)}x}]/\mathrm{(2}x)$, we have *δ*_00_ = *f*_*δ*_(*N*_00_*S*_00_, *ε*), *δ*_*j*_ = *f*_*δ*_((*N*_0*j*_ + *N*_*j*0_)*S*_*j*_,*ε*), *j* = 1, 2 and ${\delta }_{{\rm{\Delta }}}={f}_{\delta }(({N}_{11}^{{{\rm{\Delta }}}^{+}}+{N}_{11}^{{{\rm{\Delta }}}^{-}}){T}_{{\rm{\Delta }}},\,\varepsilon )$.

With the mean values $\langle {\underline{s}}_{1}^{Z}\rangle$ and $\langle {\bar{e}}_{1}^{ph}\rangle$ defined in Eqs (19) and (20), the lower bound of the yield ${\underline{s}}_{1}$ and the upper bound of the phase-flip error rata ${\bar{e}}_{1}^{ph}$ corresponding to *s*_1_ in Eq. (16) can be estimated by^29,33^

22 $${\underline{s}}_{1}=\langle {\underline{s}}_{1}^{Z}\rangle \mathrm{(1}-{\delta }_{1}^{c}),\,{\bar{e}}_{1}^{ph}=\langle {\bar{e}}_{1}^{ph}\rangle \mathrm{(1}+{\delta }_{1}^{\text{'}c}),$$

where ${\delta }_{1}^{c}={f}_{\delta }({a}_{1}{N}_{zz}^{c}\langle {\underline{s}}_{1}^{Z}\rangle ,\,\varepsilon )$ and ${\delta }_{1}^{\text{'}c}={f}_{\delta }({a}_{1}{N}_{zz}^{c}{\underline{s}}_{1}\langle {\bar{e}}_{1}^{ph}\rangle ,\,\varepsilon )$ with ${N}_{zz}^{c}=2{p}_{z}\mathrm{(1}-{p}_{z}){N}_{zz}$ and ${a}_{1}={\mu }_{z}{e}^{-{\mu }_{z}}$ being the probability to emit a single-photon state from source *ρ*_*z*_.

With the lower bound of *s*_1_ and the upper bound of ${e}_{1}^{ph}$ in Eq. (22), the final key rate can be calculated with Eq. (16).
